# Tumor heterogeneity in gastrointestinal stromal tumors of the small bowel: volumetric CT texture analysis as a potential biomarker for risk stratification

**DOI:** 10.1186/s40644-018-0182-4

**Published:** 2018-12-05

**Authors:** Cui Feng, Fangfang Lu, Yaqi Shen, Anqin Li, Hao Yu, Hao Tang, Zhen Li, Daoyu Hu

**Affiliations:** 1 Department of Radiology, Tongji Hospital, Tongji Medical College, Huazhong University of Science and Technology, 1095 Jiefang Avenue, Qiaokou District, Wuhan, 430030 China; 2 Department of Radiology, Luoyang Central Hospital, Zhengzhou University, Luoyang, 471009 China

**Keywords:** Gastrointestinal stromal tumors, Computed tomography, Pathology, Texture analysis, Risk assessment

## Abstract

**Background:**

To explore whether volumetric CT texture analysis (CTTA) can serve as a potential imaging biomarker for risk stratification of small bowel gastrointestinal stromal tumors (small bowel-GISTs).

**Methods:**

A total of 90 patients with small bowel-GISTs were retrospectively reviewed, of these, 26 were rated as high risk, 13 as intermediate risk, and 51 as low or very low risk. Histogram parameters extracted from CT images were compared among small bowel-GISTs with different risk levels by using one-way analysis of variance. Receiver operating characteristics (ROCs) and areas under the curve (AUCs) were analyzed to determine optimal histogram parameters for stratifying tumor risk.

**Results:**

Significant differences in mean attenuation, 10th, 25th, 50th, 75th and 90th percentile attenuation, and entropy were found among high, intermediate, and low risk small bowel-GISTs (*p* ≤ 0.001). Mean attenuation, 10th, 25th, 50th, 75th and 90th percentile attenuation, and entropy derived from arterial phase and venous phase images correlated significantly with risk levels (*r* = 0.403–0.594, *r* = 0.386–0.593, respectively). Entropy in venous phase reached the highest accuracy (AUC = 0.830, *p* < 0.001) for differentiating low risk from intermediate to high risk small bowel-GISTs, with a cut-off value of 5.98, and the corresponding sensitivity and specificity were 82.4 and 74.4%, respectively.

**Conclusions:**

Volumetric CT texture features, especially entropy, may potentially serve as biomarkers for risk stratification of small bowel-GISTs.

## Background

Small bowel gastrointestinal stromal tumors (small bowel-GISTs) are common mesenchymal tumors that account for almost 30% of all gastrointestinal stromal tumors (GISTs) [1]. Surgical resection is regarded as the main modality of treatment for localized GISTs. However, there is a potential risk of postoperative recurrence and metastasis for high risk GISTs within the first five years [2, 3]. The prognosis of small bowel-GISTs has been improved with the introduction of molecularly targeted agents such as imatinib [4, 5], which is recommended based on the tumor risk level for postoperative specimens according to the National Institutes of Health (NIH) consensus classification system (version 2008) [6]. Accurate evaluation of the risk level of GISTs at diagnosis is so important, as it has a great influence on the treatment selection and prognosis assessment [5]. However, it is difficult to obtain reliable evidence for risk stratification of unresectable small bowel-GISTs in clinical practice.

Unlike gastric GISTs, small bowel-GISTs are difficult to diagnose and characterize via endoscopic biopsy. Furthermore, biopsy can increase the risk of tumor seeding [7]. Cross-sectional imaging, especially computed tomography (CT), is currently the main imaging modality used to provide information on small bowel-GISTs at their initial presentation [8]. The diagnosis of small bowel-GISTs by conventional CT is based mainly on tumor size, enhancement characteristics, and the site of origin [9, 10]. But, tumor risk cannot be accurately assessed based on conventional morphological analysis.

Better methods of tumor risk stratification in patients with small bowel-GISTs are still needed. By evaluating the distribution of tissue gray-level on CT images, CT texture analysis (CTTA) performed on either the largest cross-section or whole tumour datasets can be used to assess tumor heterogeneity quantitatively [11–14]. Compared with single-section analysis, CTTA performed on whole tumor datasets may be more representative and repeatable [15]. Recently volumetric CTTA has been applied to a variety of tumors, including lung carcinoma and colorectal cancer, demonstrating that texture features were highly associated with 5-year survival [16, 17].

In a study of 78 patients with GISTs, CTTA has been proved to have the potential to predict malignant risk [18]. While, in this study, most of the GISTs were located in the stomach, which may cause some bias. To date, few studies have utilized CTTA to predict the risk level of small bowel-GISTs. Therefore, the present study was designed to explore the potential value of volumetric CTTA in the risk stratification of small bowel-GISTs.

## Methods

### Study population

**Fig. 1:**
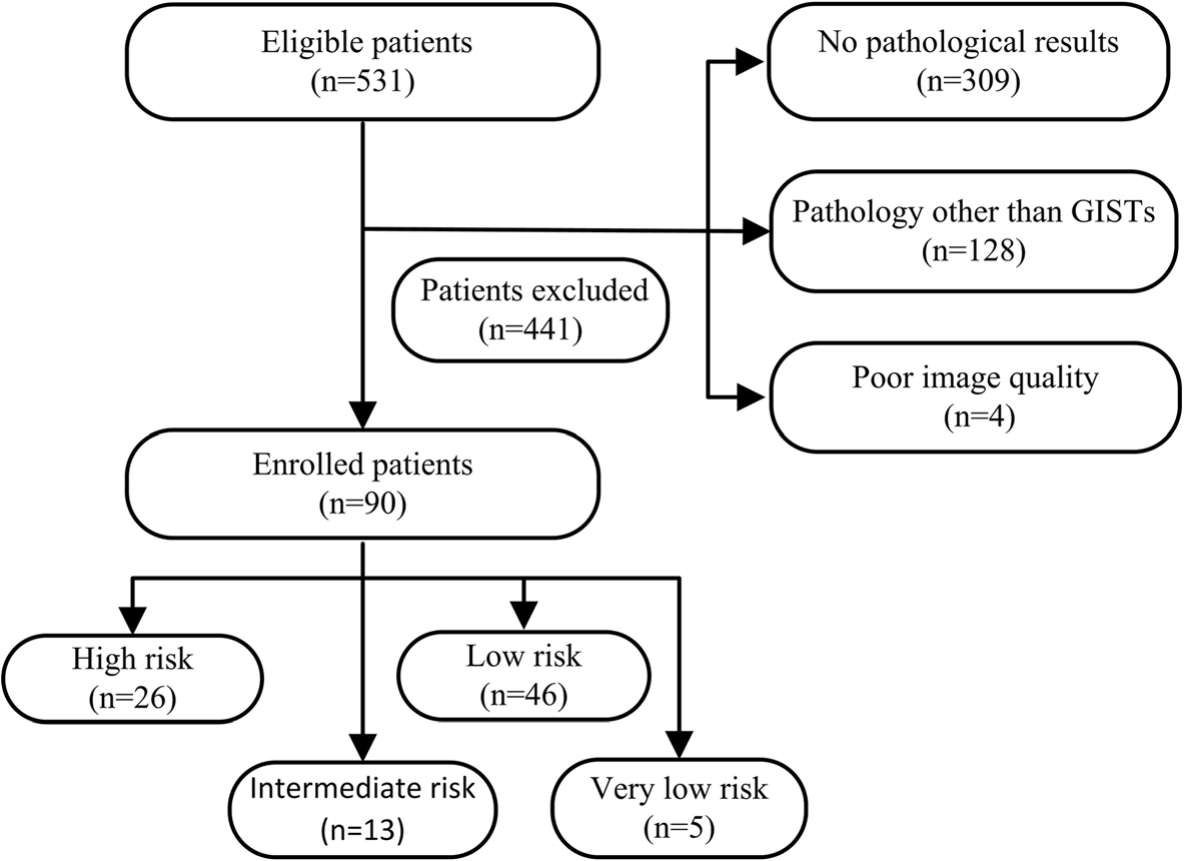
Flowchart of the study population

This retrospective study was approved by our institutional review board, and patient informed consent was waived. In total 531 patients clinically suspected of having small bowel-GISTs were enrolled from March 2012 to March 2016. Inclusion criteria were as follows: (1) previous surgery and histopathology confirmed primary small bowel-GISTs; (2) there was no treatment prior to the CT examination; (3) CT resulted in adequate image acquisition and good image quality. Exclusion criteria were as follows: (a) unresectable small bowel-GISTs (*n* = 309); (b) pathology other than GISTs (*n* = 128); (c) inadequate image quality (*n* = 4). Finally, 90 patients (32 female, 58 male; mean age, 53.2 ± 9.4 years; age range, 26–71 years) with histopathologically proved primary small bowel-GISTs were included. According to the modified version of the NIH criteria proposed by Joensuu [6] in 2008, risk stratification was assessed by two pathologists based on tumor size (maximum diameter), mitotic rate (the number of mitoses per 50 high-power fields), primary tumor site, and tumor rupture. Enrolled patients were divided into 4 categories: high, intermediate, low, and very low risk. A flowchart of the study population is shown in Fig. 1.

### Image acquisition

To dilate the bowel, we chose 20% *w*/*v* mannitol as the oral contrast agent in our study. The solution was prepared by diluting 250 mL of mannitol into 1750 mL of water. Patients were instructed to drink 1500–2000 mL over 40–60 min prior to the CT scanning in portions of 300–500 mL each every 10 min. The patients were also required to fast for 6 h before the procedure.

All patients—placed supine, feet-first on the CT table—underwent dual phase contrast-enhanced CT using a 64-slice multidetector CT (MDCT) scanner (Discovery CT750 HD, GE Healthcare, WI, USA). Intravenous contrast medium 370 mg I/mL iopromide (Ultravist 370, Bayer Schering Pharma, Berlin, Germany) was administered at a flow rate of 3.5 mL/s, followed by a 20 mL saline flush. The total contrast volume was 1.5 mL/kg. Contrast material was injected through the antecubital vein with an 18 gauge intravenous cannula using a dual-head injector, each with an injection time of 20 s. Arterial phase scanning started at 6 s after a threshold enhancement of the abdominal aorta reached 120 HU, monitoring by using a bolus tracking technique (Smartprep, GE Healthcare Technologies). Venous phase scanning was initiated at 25 to 30 s after the completion of the arterial phase scanning.

The CT imaging parameters were as follows: automatic tube current; tube voltage,120 kV; rotation time, 0.5 s; detector pitch, 0.984:1; matrix, 512 × 512; table speed, 39.37 mm/rotation; and slice thickness/interval, 5 mm.

### Image processing

The data were measured by two board-certified abdominal radiologists (F.C and L.Z, with 8 and 18 years of experience in abdominal imaging, respectively), who were blinded to the histologic results. The regions of interest (ROIs) were manually delineated along the edge of each lesion on axial images (slice thickness, 2 mm), excluding adjacent blood vessels, normal bowel wall, and contents. The ROI of each layer was fused to obtain whole tumor volume voxel information. Texture features were automatically extracted and calculated by using the software program (CT Kinetics, GE Healthcare, WI, USA). Texture parameters derived from CT images were as follows: mean attenuation; 10th, 25th, 50th, 75th and 90th percentile attenuation; kurtosis (magnitude of pixel distribution); skewness (asymmetry of pixel histogram); entropy (the irregularity of pixel distribution). All image processing was performed separately for arterial and venous phase CT images. The average value of the two measurements was regarded as the parameter value for each lesion.

### Statistical analysis

Statistical analysis was performed by using SPSS software (version 17.0 for Windows; SPSS, Chicago, IL). Continuous variables were expressed as mean ± standard deviation, and categorical variables were expressed as frequency (percentage). The data normality was evaluated with the Kolmogorov-Smirnov test. According to the results of the normal distribution test, one-way analysis of variance was performed for comparisons of CT histogram parameters among different risk levels of small bowel-GISTs, followed by Bonferroni test for post hoc pairwise comparisons. Correlations between histogram parameters and risk levels were analyzed by using Spearman rank correlation. *p* < 0.05 was considered statistically significant. ROCs were used to determine the diagnostic accuracy of histogram parameters for differentiating low-risk from intermediate to high risk small bowel GISTs. AUCs, sensitivity, and specificity were estimated to determine the optional parameter. Interobserver agreement of the two readers for each parameter was assessed by calculating intraclass correlation coefficient (ICC).

## Results

### Patient characteristics and histologic findings

The clinical and pathologic data are summarized in Table 1. To balance the numbers in each group, patients at very low risk were assigned to the low risk group. The maximal tumor diameter ranged from 1.2 to 15 cm (mean diameter, 4.8 cm).

**Table 1:** Patient characteristics

Characteristics	Number (%)
Gender	
Male	58 (64.4)
Female	32 (35.6)
Age (years)	53 ± 9 (26–71)^*^
Tumor risk	
High	26 (28.9)
Intermediate	13 (14.4)
Low	46 (51.1)
Very low	5 (5.6)
Primary mass location	
Duodenum	28 (31.1)
Jejunum	51 (56.7)
Ileum	6 (6.7)
Jejunoileum junction	5 (5.6)
Maximum diameter	
≤ 2 cm	10 (11.1)
> 2 cm to ≤5 cm	53 (58.9)
> 5 cm to ≤10 cm	21 (23.3)
> 10 cm	6 (6.7)

Note: ^*^ Data is presented as mean ± standard deviation (range)

### Comparison of CT histogram parameters

Table 2 summarizes CT histogram parameters among high, intermediate, and low risk small bowel-GISTs. The values for mean attenuation, 10th, 25th, 50th, 75th and 90th percentile attenuation, and entropy were significantly different (*p* ≤ 0.001) among different risk levels, and the values decreased from low to high risk small bowel-GISTs both in arterial and venous phases. However, no significant differences in skewness and kurtosis were detected among high, intermediate, and low risk small bowel-GISTs (*p* > 0.05 for all).

**Table 2:** The comparisons of histogram parameters among high, intermediate and low risk small bowel-GISTs

Parameters	Arterial phase				Venous phase			
	Low risk (*n* = 51)	Intermediate risk (*n* = 13)	High risk (*n* = 26)	*p* value	Low risk (n = 51)	Intermediate risk (n = 13)	High risk (n = 26)	*p* value
Mean attenuation (HU)	90.51 ± 25.36	78.69 ± 25.13	63.59 ± 18.26^**‡**^	< 0.001*	93.14 ± 23.50	80.16 ± 19.60	69.38 ± 18.97^**‡**^	< 0.001*
10th percentile attenuation (HU)	55.11 ± 26.59	39.40 ± 28.97	26.56 ± 18.70^**‡**^	< 0.001*	60.27 ± 26.88	44.50 ± 25.99	31.68 ± 21.17^**‡**^	< 0.001*
25th percentile attenuation (HU)	71.42 ± 26.37	57.78 ± 26.63	43.53 ± 18.68^**‡**^	< 0.001*	76.00 ± 25.53	61.54 ± 22.73	49.69 ± 20.51^**‡**^	< 0.001*
50th percentile attenuation (HU)	89.68 ± 25.96	78.07 ± 25.10	62.65 ± 18.87^**‡**^	< 0.001*	93.34 ± 23.16	80.56 ± 19.46	69.61 ± 19.36^**‡**^	< 0.001*
75th percentile attenuation (HU)	108.99 ± 27.09	98.80 ± 24.46	82.83 ± 19.52^**‡**^	< 0.001*	110.36 ± 19.12	100.68 ± 16.03	87.62 ± 14.22^**‡**^	< 0.001*
90th percentile attenuation (HU)	126.92 ± 28.80	118.41 ± 25.26	100.78 ± 20.59^**‡**^	< 0.001*	125.65 ± 23.14	115.57 ± 17.72	106.15 ± 19.91^**‡**^	0.001*
Skewness	0.15 ± 0.26	0.14 ± 0.17	0.22 ± 0.24	0.488	−0.09 ± 0.23	−0.06 ± 0.07	0.01 ± 0.28	0.272
Kurtosis	3.16 ± 0.41	3.32 ± 0.21	3.35 ± 0.54	0.121	3.14 ± 0.50	3.09 ± 0.12	3.09 ± 0.40	0.909
Entropy	6.08 ± 0.44	5.82 ± 0.36	5.48 ± 0.39^**†‡**^	< 0.001*	6.20 ± 0.37^**†**^	5.93 ± 0.37	5.70 ± 0.340^**‡**^	< 0.001*

Note: Data are presented as mean ± SD; HU = Hounsfield unit

* *p* < 0.05 with One-Way Analysis of Variance

Post hoc subgroup comparisons: † *p* < 0.05 vs. intermediate risk group, ‡ *p* < 0.05 vs. low risk group

With regard to pairwise comparisons, the entropy value was the highest for low risk small bowel-GISTs as 6.08 ± 0.44 (for low risk vs. intermediate risk, *p* = 0.061; for low risk vs. high risk, *p* < 0.001) in arterial phase, followed by intermediate risk small bowel-GISTs as 5.82 ± 0.36 (for intermediate risk vs. high risk, *p* = 0.025). Meanwhile, the entropy value also reached the highest for low risk small bowel-GISTs as 6.20 ± 0.37 (for low risk vs. intermediate risk, *p* = 0.018; for low risk vs. high risk, *p* < 0.001) in venous phase, followed by intermediate risk small bowel-GISTs as 5.93 ± 0.37 (for intermediate risk vs. high risk, *p* = 0.096).

The interobserver agreement between the two readers was excellent for mean attenuation, 10th, 25th, 50th, 75th, and 90th percentile attenuation, skewness, kurtosis, and entropy in our study cohort (ICC, 0.923–0.999).

### Correlations between histogram parameters and risk levels

Correlations between histogram parameters and risk levels are summarized in Table 3. Mean attenuation, 10th, 25th, 50th, 75th and 90th percentile attenuation, and entropy correlated significantly with the risk levels of small bowel-GISTs derived from arterial venous CT images (*r* = 0.403–0.594; all *p* < 0.001), and those derived from venous phase CT images (*r* = 0.386–0.593; all *p* < 0.001). But, skewness and kurtosis correlated negatively with the risk levels.

**Table 3:** The correlations of histogram parameters with the risk levels of small bowel-GISTs

Parameters	Arterial phase		Venous phase	
	*r*	*p* value	*r*	*p* value
Mean attenuation (HU)	0.455	< 0.001^*^	0.461	< 0.001^*^
10th percentile attenuation (HU)	0.497	< 0.001^*^	0.476	< 0.001^*^
25th percentile attenuation (HU)	0.473	< 0.001^*^	0.468	< 0.001^*^
50th percentile attenuation (HU)	0.455	< 0.001^*^	0.447	< 0.001^*^
75th percentile attenuation (HU)	0.426	< 0.001^*^	0.529	< 0.001^*^
90th percentile attenuation (HU)	0.403	< 0.001^*^	0.386	< 0.001^*^
Skewness	− 0.099	0.354	− 0.090	0.399
Kurtosis	−0.192	0.070	−0.077	0.470
Entropy	0.594	< 0.001^*^	0.593	< 0.001^*^

Note: *p* < 0.05 with Spearman correlation analysis

### Diagnostic performance of histogram parameters for risk stratification

**Fig. 2:**
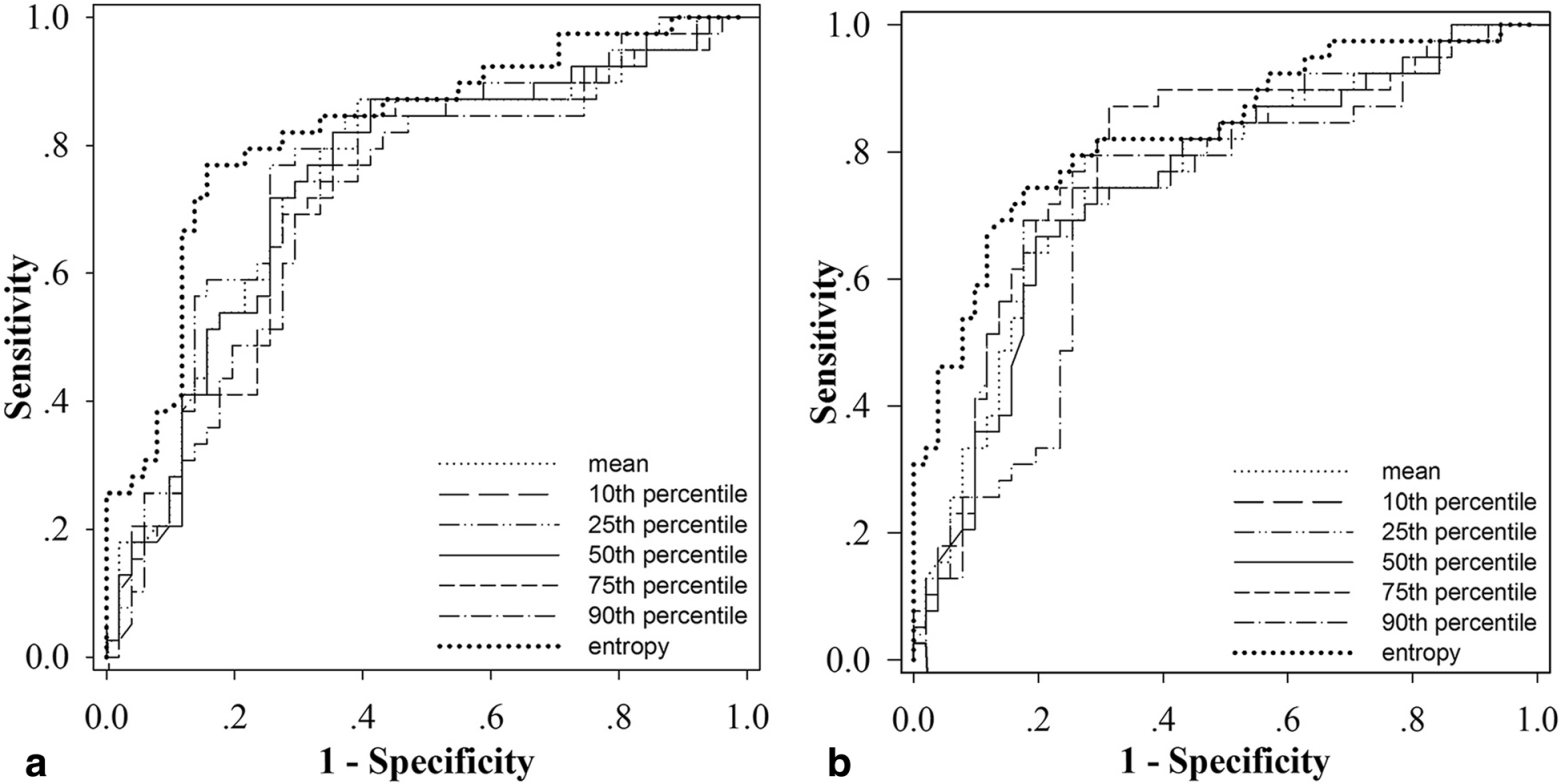
ROC curves for mean attenuation, median attenuation, 10th, 25th, 50th, 75th, and 90th percentile attenuation and entropy in differentiating low risk small bowel-GISTs from intermediate to high risk small bowel-GISTs in arterial phase (**a**) and venous phase (**b**)

Analysis of ROC curves and diagnostic performance is shown in Table 4 and Fig. 2.

**Table 4:** The diagnostic performance for differentiating low risk from intermediate to high risk small bowel-GISTs

Parameters	Arterial phase					Venous phase				
	AUC	Cut-off	Sensitivity (%)	Specificity (%)	*p* value	AUC	Cut-off	Sensitivity (%)	Specificity (%)	*p* value
Mean attenuation (HU)	0.747	78.88	66.7	79.5	< 0.001^*^	0.754	81.92	72.5	74.4	< 0.001^*^
10th percentile attenuation (HU)	0.778	36.96	82.4	66.7	< 0.001^*^	0.766	46.18	74.5	71.8	< 0.001^*^
25th percentile attenuation (HU)	0.761	57.97	74.5	76.9	< 0.001^*^	0.760	60.41	82.4	69.2	< 0.001^*^
50th percentile attenuation (HU)	0.746	79.90	64.7	82.1	< 0.001^*^	0.746	74.71	80.4	66.7	< 0.001^*^
75th percentile attenuation (HU)	0.726	104.29	58.8	84.6	< 0.001^*^	0.787	104.62	68.6	87.2	< 0.001^*^
90th percentile attenuation (HU)	0.712	112.57	66.7	74.4	0.001^*^	0.710	116.48	72.5	79.5	0.001^*^
Entropy	0.823	5.86	82.4	76.9	< 0.001^*^	0.830	5.98	82.4	74.4	< 0.001^*^

Note: AUC = area under the curve, HU = Hounsfield unit

* *p* < 0.05

**Fig. 3:**
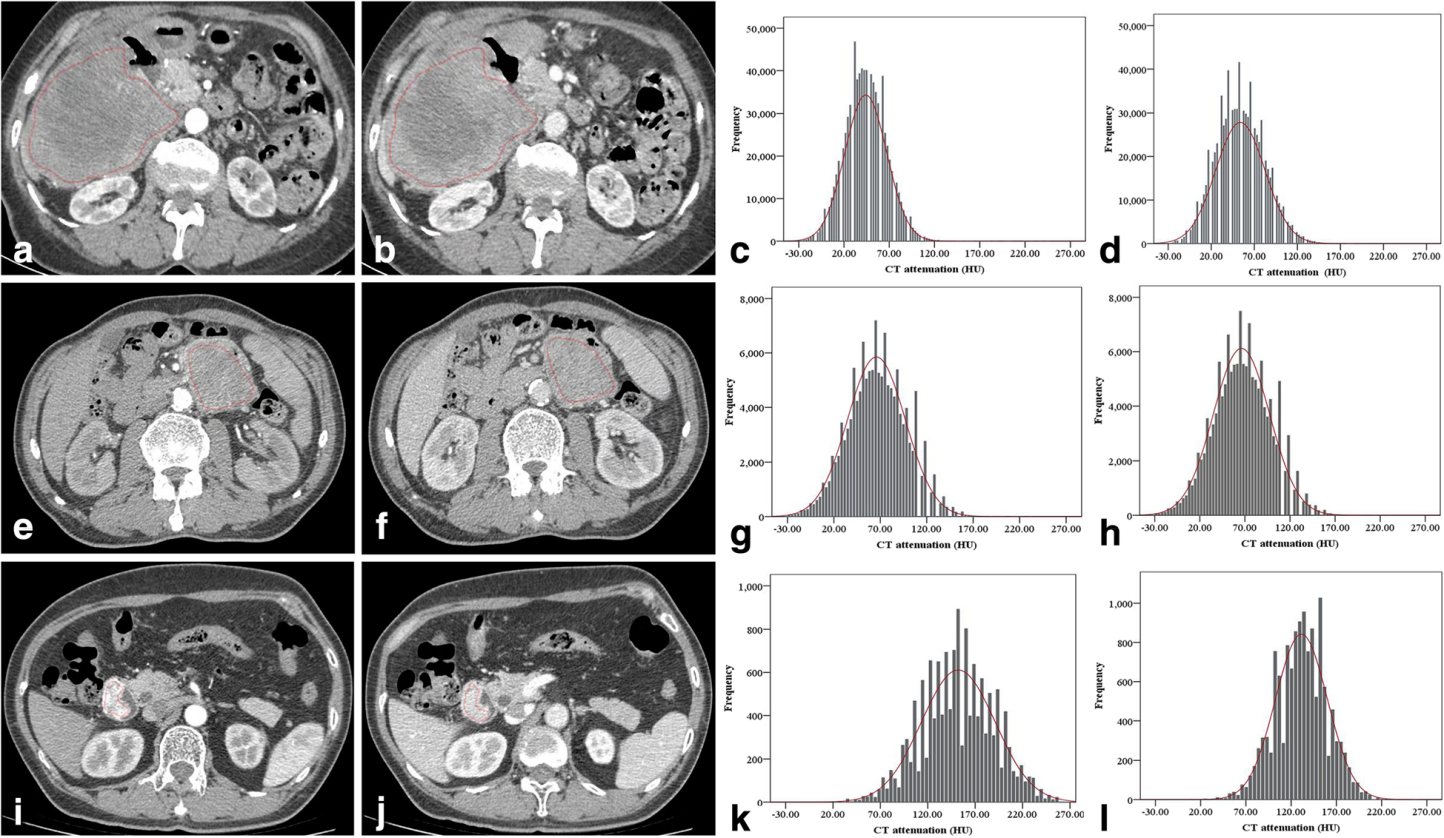
CT texture analysis of small bowel-GISTs with different risk levels. CT images of high risk in arterial phase (**a**) and venous phase (**b**) show an irregular external growth mass situated in the right upper abdomen with heterogeneous enhancement in a 66-year-old female. Histograms in arterial phase (**c**) and venous phase (**d**) show lower distribution of CT values. Mean attenuation and entropy were 47.00HU and 5.23 in arterial phase, 57.64HU and 5.39 in venous phase, respectively. CT images of intermediate risk in the arterial phase (**e**) and venous phase (**f**) show a rounded external mass at the proximal jejunum in a 60-year-old male. Histograms in arterial phase (**g**) and venous phase (**h**) show the distribution of CT values. Mean attenuation and entropy were 68.72HU and 5.31 in arterial phase, 70.22HU and 5.66 in venous phase, respectively. CT images of low risk in the arterial phase (**i**) and venous phase (**j**) show an irregular internal mass with obvious enhancement located at the descending duodenum in a 60-year-old female. Histograms in arterial phase (**k**) and venous phase (**l**) show higher distribution of CT values. Mean attenuation and entropy were 155.15HU and 6.21 in arterial phase, 133.26HU and 6.14 in venous phase, respectively

In arterial phase, entropy achieved the highest accuracy (AUC, 0.823; 95% CI: 0.734, 0.912) for differentiating intermediate to high risk from low risk small bowel-GISTs, with a cut-off value of 5.86, the corresponding sensitivity and specificity were 82.4 and 76.9%, respectively. Meanwhile, entropy also had the highest accuracy (AUC, 0.830; 95% CI: 0.743, 0.917) in the venous phase, with a cut-off value of 5.98, the corresponding sensitivity and specificity were 82.4 and 74.4%, respectively. Representative cases of small bowel-GISTs with different risk levels are presented in Fig. 3.

## Discussion

Volumetric CTTA has recently been acknowledged as a promising tool allowing for the quantification of spatial intratumor heterogeneity by assessing the distribution of gray-level [14]. Several previous studies have suggested that CTTA may be of value in evaluating clinical stage, pathologic grade, and prognosis in various types of gastrointestinal tumors, including esophageal, gastric, and colorectal cancers [11, 12, 17, 19]. However, the application of CTTA in predicting the outcome of small bowel-GISTs has not already been reported.

In the present study, significant differences in mean attenuation, 10th, 25th, 50th, 75th and 90th percentile attenuation, and entropy were found among high, intermediate, and low risk small bowel-GISTs in both arterial and venous phases. Entropy derived from the venous phase images reached the highest accuracy with an AUC of 0.830 for differentiating low risk from intermediate to high risk small bowel-GISTs.

Our data showed that mean attenuation, 10th, 25th, 50th, 75th and 90th percentile attenuation, and entropy correlated significantly with the risk levels, and the values decreased from low to high risk small bowel-GISTs. CT attenuation represents the degree of tumor enhancement, as previous studies have reported [12], and higher attenuation probably reflects the higher vascularity that characterizes more aggressive tumors. Zhou et al. [9] also reported that these enhancement characteristics were associated with the risk level of GISTs; that is, the higher the tumor risk level, the more noticeable the enhancement. The explanation for this contradictory result may be that our study analyzed texture parameters based on entire tumors instead of a single axial level without excluding the necrotic components when selecting ROI. It has been reported that small bowel-GISTs were hypervascular tumors, and that the higher the risk level, the more prone a tumor would be to necrosis [7, 8, 20]. In other words, the development of intratumoural necrosis could reduce CT attenuation value of whole tumors.

With regard to subgroup analysis, our results revealed that there was difference in entropy between arterial and venous phases analysis. Similar results were reported by Liu et al. [18], they evaluated 78 patients with GISTs and found there were significant differences of CT texture parameters at different malignancy risks between arterial and venous phases. Previous studies have indicated that CT texture analysis can predict the histopathologic characteristics of gastric cancers [19, 21, 22]. Liu et al. reported that the differential invasiveness of tumors of different grades depended mainly on neovascularization, which can be evaluated by contrast-enhanced CT attenuation [19]. The possible reason may be that the enhancement characteristics of the arterial phase reflect the blood supply, whereas the characteristics of the venous phase reflect the distribution of the contrast agent in interstitial space, accounting for the differences in performance between the arterial and venous phases. This suggests that entropy correlate with the risk level of small bowel-GISTs and may be helpful in the prognostic assessment of small bowel-GISTs.

Entropy represents the irregularity of gray-level distribution, which is associated with tumor heterogeneity caused by necrosis, angiogenesis, and cellular density [23]. Some previous studies have reported that higher entropy represented higher tumor aggressiveness and poorer prognosis [24, 25]. Liu et al. [18] also reported that the entropy extracted from venous phase images correlated with the risk level of GISTs significantly, distinguishing low from very low risk level with an AUC of 0.684. Conversely, our data indicate that lower entropy is significantly correlated with higher risk, and that entropy is the optimal parameter for distinguishing different risk levels with an AUC from 0.719 to 0.887, reflecting a negative correlation with tumor risk level. Similar conclusions have been reported in the study of Ng et al. [17], in which they demonstrated that lower entropy was associated with a poorer prognosis in colorectal tumors. Hence, there is a discrepancy between entropy, heterogeneity, and the assessment of different tumor types. The reasons for these differences are not clear, a study has reported that they may be related to the differences in ROI selection methods (whole tumor/a single axial level) [26]. Another possible reason may be that there were differences in texture features between contrast-enhanced and unenhanced CT images. Ng et al. [17] assumed that a higher tumor grade demonstrated higher vascular permeability, leading to smaller differences in the distribution of contrast between vessels and adjacent parenchyma on contrast-enhanced CT and less heterogeneity in texture analysis. This remains a somewhat controversial area and warrants additional investigation in the future.

Higher skewness and lower kurtosis were also significantly associated with the presence of a K-ras mutation in non-small cell lung cancer in the study of Weiss et al. [27] However, in the present study, there was no statistically significant difference in skewness and kurtosis between different tumor risk levels, reflecting a limited role of skewness and kurtosis in the risk stratification of small bowel-GISTs.

The present study has several limitations. First, it was a retrospective single-center study with inherent biases in patient selection. In a single-institution study, bias was unavoidable because of the patient cohort and the nature of the study. We analyzed only the diagnostic capability of texture analysis without considering the assessment of treatment and prognosis; hence, long-term follow-up would be needed to strengthen our initial findings. Third, the number of patients with very low risk small bowel-GISTs was relatively small. Therefore, to balance the numbers in each group, those at very low risk were grouped together with those at low risk. Because few related studies have been reported, hence, further studies with a larger sample size will be needed.

## Conclusions

In conclusion, volumetric CT texture parameters, especially entropy derived from venous phase, may be potential biomarkers serving to stratify the risk of small bowel-GISTs. This might improve assessment before the initiation of treatment and optimize treatment programs for these patients.

## Abbreviations

AUC: Area under the ROC curve
CTTA: Computed tomography texture analysis
GISTs: Gastrointestinal stromal tumours
HU: Hounsfield unit
ICC: Intraclass correlation coefficient
MDCT: Multidetector computed tomography
NIH: National Institutes of Health
ROC: Receiver operating characteristic
ROI: Region of interest
SD: Standard deviation
